# Synthesis and Applications of Supramolecular Flame Retardants: A Review

**DOI:** 10.3390/molecules28145518

**Published:** 2023-07-19

**Authors:** Simeng Xiang, Jiao Feng, Hongyu Yang, Xiaming Feng

**Affiliations:** College of Materials Science and Engineering, Chongqing University, Shapingba, Chongqing 400044, China; 202109021141t@stu.cqu.edu.cn (S.X.); 202109021177t@cqu.edu.cn (J.F.)

**Keywords:** supramolecular flame retardants, sustainability, fire safety, polymers, mechanical properties

## Abstract

The development of different efficient flame retardants (FRs) to improve the fire safety of polymers has been a hot research topic. As the concept of green sustainability has gradually been raised to the attention of the whole world, it has even dominated the research direction of all walks of life. Therefore, there is an urgent calling to explore the green and simple preparation methods of FRs. The development of supramolecular chemistry in the field of flame retardancy is expanding gradually. It is worth noting that the synthesis of supramolecular flame retardants (SFRs) based on non-covalent bonds is in line with the current concepts of environmental protection and multi-functionality. This paper introduces the types of SFRs with different dimensions. SFRs were applied to typical polymers to improve their flame retardancy. The influence on mechanical properties and other material properties under the premise of flame retardancy was also summarized.

## 1. Introduction

**Figure 1:**
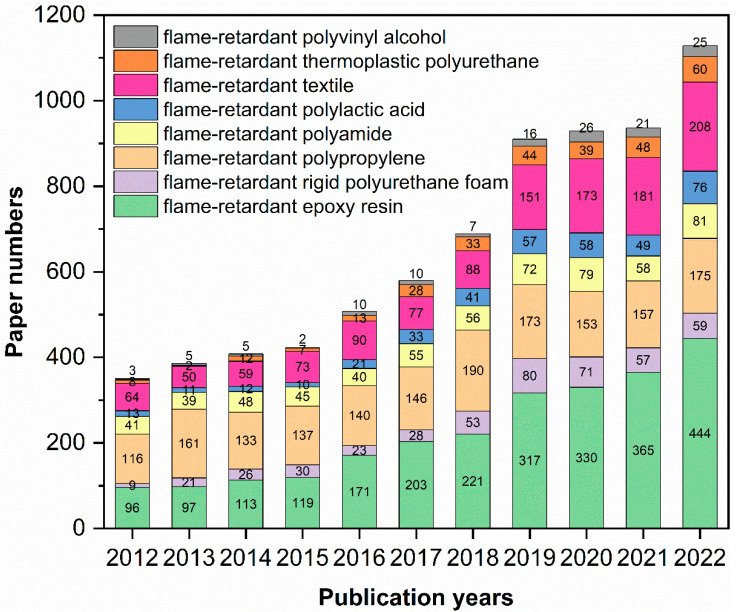
Paper publications of flame-retardant typical polymers from 2012 to 2022. (Data from Web of Science, as of December 2022).

With the development of science and technology, the application of polymer materials has penetrated all aspects of our life and production, making existence more convenient [1,2]. At the same time, there are potential risks. Because most polymer materials are rich in carbon, hydrogen, and other elements, their intrinsic molecular structure determines the combustibility or flammability. They may decompose and burn at high temperatures, causing fires [3,4]. In the new situation of consumption upgrading and the rapid-development of emerging industries, higher requirements are put forward for the performance of polymers. Improving the flame retardancy of polymers can improve the reliability and application fields of their products (such as new energy vehicles [5], electronics and electrical products [6,7], and aerospace products [8]). In addition, Figure 1 shows the number of scientific research publications on flame retardancy of some typical polymers in the past decade. The increasing trend also reflects that the research of flame-retardant polymers is challenging and developmental.

Flame retardants (FRs), as additives, are applied to polymer materials. They achieve flame retardancy mainly in the condensed phase and/or gas phase. In the past, halogenated FRs were the mainstay. However, in practical application, some halogen FRs will release harmful substances (such as corrosive hydrogen halide gas and toxic carcinogens dioxins and furans) during thermal decomposition, which will undoubtedly cause great harm to human health and the ecological environment [9,10]. Meanwhile, some laws and regulations also put forward explicit requirements for the use of FRs. In 2013, the global ban on hexabromocyclododecane was proposed by the United Nations Environment Programme in the Stockholm Convention on Persistent Organic Pollutants [11]. In 2019, the European Union issued regulations on the prohibition of halogen FRs in electronic displays [12]. In 2022, New York State amended the control content of FRs in upholstered furniture, mattresses, electronic display housings, supports, etc., in the Bill (S4630B/A5418B) [13]. Today, with the gradual deepening of environmental awareness, environmental-friendly halogen-free FRs with no (low) toxicity, low smoke, and low corrosion [9,14,15,16] are being developed. Statistically, halogen-free flame-retardant products dominated the market in 2020 (up from 59% share) [17]. Among them, phosphorus FRs [18,19], nitrogen FRs [20,21], phosphorus–nitrogen FRs [22,23,24,25], and metal-compound FRs [26,27,28] have been widely studied. In the synthesis and development of FRs, it is found that a particular status does not conform to the concept of green and sustainable development. Because some synthesis conditions are relatively harsh (such as high temperature, high pressure, and inert environment) and the synthesis procedure is relatively complex, some involve toxic and harmful organic solvents, such as trichloromethane, ether, acetone, tetrahydrofuran, and acetonitrile [29,30,31,32]. This brings lots of trouble to the subsequent processing. It also burdens the environment greatly. Therefore, preparing the high-efficiency FRs in a simple, safe, and environmentally friendly manner has become an urgent focus.

Supramolecular chemistry usually refers to the combination of two (or more) molecules with specific structures and properties by non-covalent intermolecular interactions (such as ion attraction, ion-dipole interaction, dipole-dipole interaction, hydrogen bonding, and electrostatic interaction) [33]. Supramolecular chemistry is at the forefront of scientific development, which has been extensively developed in biomedicine [34], photoelectric materials [35], self-healing materials [36], and binders [37]. In the field of flame retardancy, supramolecular chemistry is also gradually expanding. It is noteworthy that supramolecular-assembly has the characteristics of easy synthesis and greenness. At present, there is no systematic review on the research progress and application of supramolecular flame retardants (SFRs) in the field of flame retardancy. This work mainly introduces the types of SFRs and their applications in the typical polymer materials. Under the premise of flame retardancy, the improvement of polymer materials in other properties by SFRs is also concerned.

## 2. Synthesis of Supramolecular Flame Retardants

**Figure 2:**
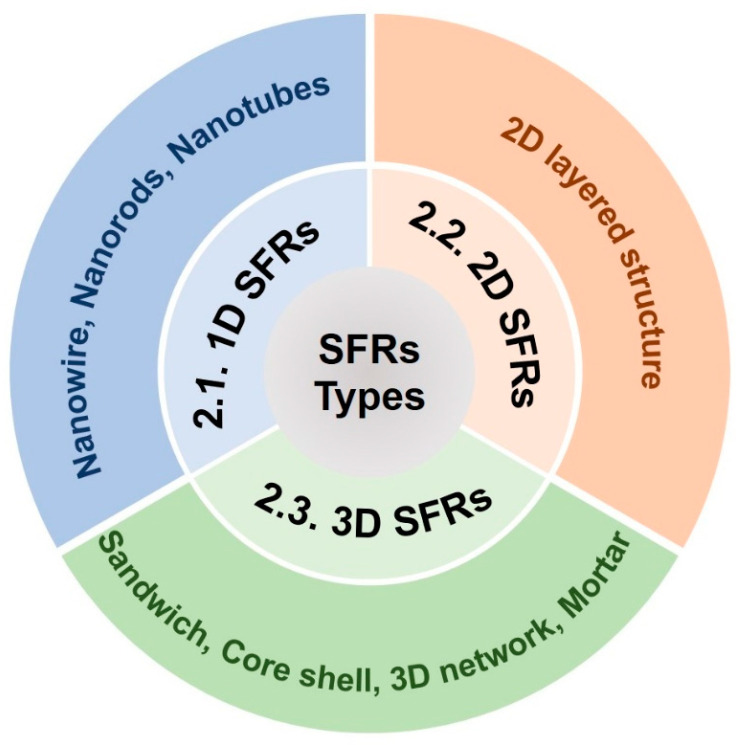
Classification of SFRs and corresponding structural types.

According to the morphological structure of FRs, FRs synthesized based on supramolecular self-assembly are classified into one-dimensional supramolecular flame retardants (1D SFRs), two-dimensional supramolecular flame retardants (2D SFRs) and three-dimensional supramolecular flame retardants (3D SFRs) (Figure 2).

### 2.1. One-Dimensional Supramolecular Flame Retardants

Some inorganic materials with 1D nanostructures, such as halloysite nanotubes (HNTs) [38,39], multiwalled carbon nanotubes (MWCNTs) [40], various whiskers [41,42], nanowire materials [43], etc., have certain flame-retardant and smoke-suppressing effects. But there are problems with agglomeration, which deteriorates the mechanical properties of the matrix due to excessive addition. How to design and optimize the more advantageous 1D organic-inorganic hybrid FRs has certain research significance for the development of efficient flame-retardant systems and improving the mechanical properties of the matrix. Currently, some studies have used such 1D inorganic nanomaterials as the building blocks and selected some classical flame-retardant phosphorus–nitrogen sources for controllably encapsulating the blocks by self-assembly to construct 1D organic-inorganic hybrid SFRs [44,45,46].

**Figure 3:**
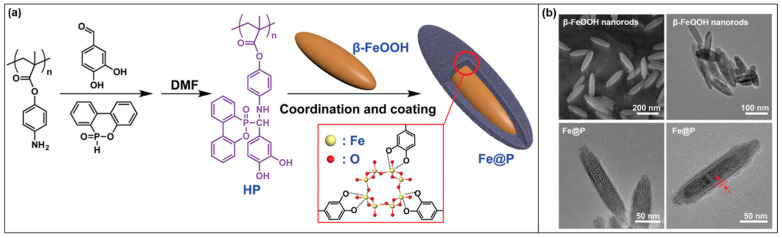
(**a**) The process of modifying β-FeOOH. (**b**) SEM images before and after modification of β-FeOOH [44].

Ting Chen et al. [44] reported a supramolecular nanorod with a core-shell structure. Covalent polymers (named HP) of flame-retardant phosphorus and nitrogen sources were firstly assembled by the Kabachnik-Fields reaction. β-FeOOH can play an effective role in smoke suppression. β-FeOOH has a tetragonal crystal system structure, and Fe^3+^ is located in the voids of octahedra. Using this unique structure, it formed an organic-inorganic hybrid by coordination with HP containing polyphenolic structures (Figure 3a). SEM showed that HP was encapsulated on the surface of spindle-like β-FeOOH nanorods (Figure 3b).

**Figure 4:**
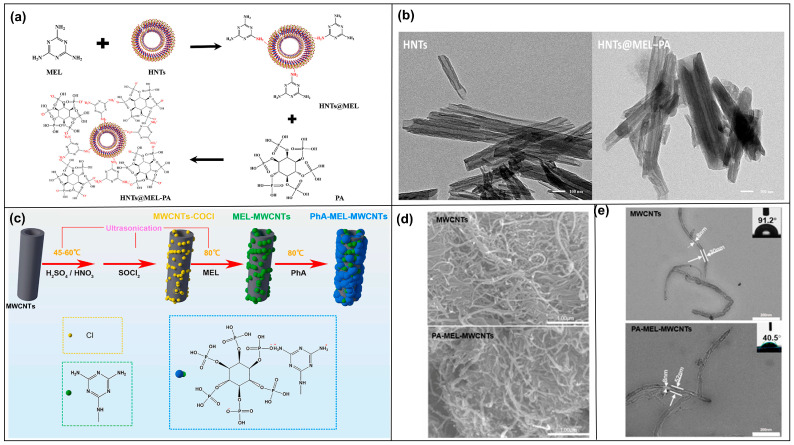
(**a**) The process of modifying HNTs. (**b**) SEM images before and after modification of HNTs [45]. (**c**) Grafting procedure of MWCNTs. (**d**) SEM images before and after grafting of MWCNTs. (**e**) Water contact Angles before and after grafting of MWCNTs [46].

Sheng Shang et al. [45] used HNTs with surface rich in active sites (e.g., Al-OH, Si-O functional groups) as the building blocks. Firstly, melamine (MEL) was modified on the surface of HNTs in aqueous solution based on hydrogen bonding. The supramolecular self-assembly was then performed by means of hydrogen bonding and ionic attraction between the phosphate group of phytic acid (PA) and the amino group of MEL (Figure 4a). A flame-retardant functionalized modified nanotube structure was successfully prepared (Figure 4b). In addition to HNTs as substrates for self-assembly, MWCNTs have also been chosen as the building blocks [46]. MEL and PA were grafted onto the surface of MWCNTs using ionic interactions and π-π stacking by successive ultrasonic stirring in aqueous solution at 80 °C (Figure 4c). It was observed that the grafted MWCNTs changed from smooth to rough (Figure 4d). Moreover, the polarity of MEL-PA makes its water contact angle smaller, indicating that its wettability improves (Figure 4e).

### 2.2. Two-Dimensional Supramolecular Flame Retardants

Currently, the construction of 1D SFRs mainly relies on the 1D structure of the selected building blocks. The diversity and additivity of supramolecular self-assembly interactions should be fully utilized. More options in assembly materials can be available to expand supramolecular assemblies from 1D to 2D. 2D SFRs exhibit a 2D lamellar form, which can affect the transfer of air, combustible volatile substances and heat, thus achieving a barrier effect. In addition, such structures tend to have a high aspect ratio and can be used to enhance the mechanical properties of the matrix [47,48,49,50].

**Figure 5:**
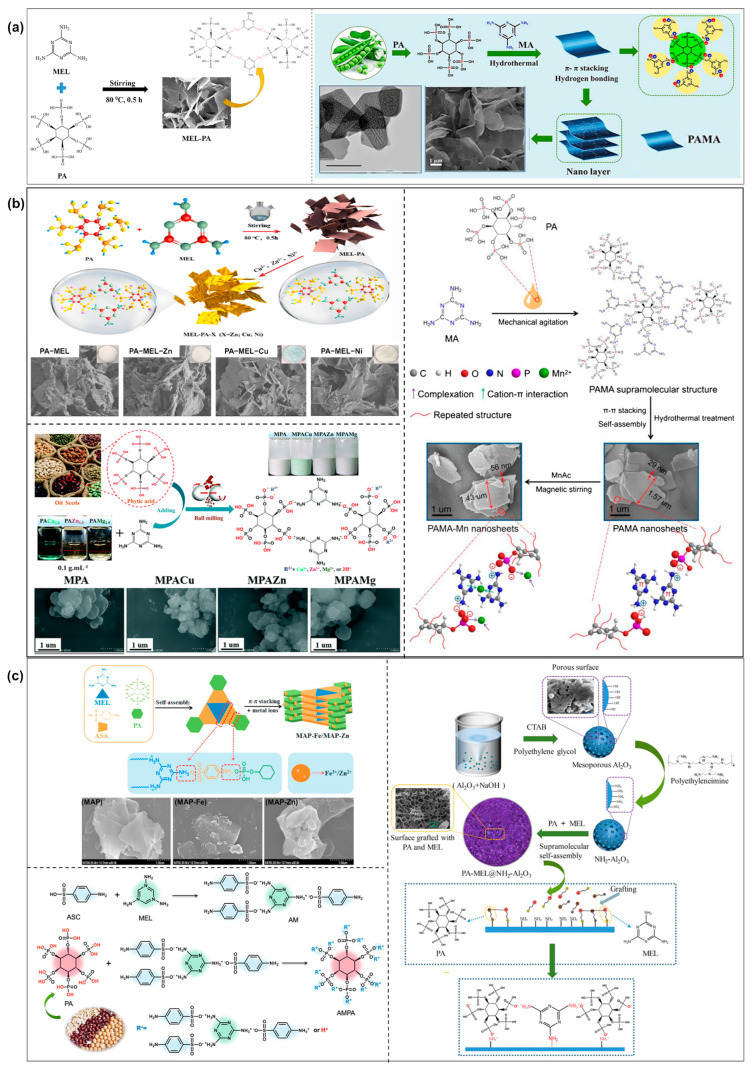
(**a**) Supramolecular self-assembly process of MEL and PA [54,55]. (**b**) Different metal ions were grafted on the basis of MEL-PA [56,57,62]. (**c**) Modify different organic or inorganic compounds on the basis of MEL-PA [58,60,61].

PA is a biomass-based flame-retardant monomer, mainly extracted from plant seeds. The structure of PA is that there are six phosphate groups in the inositol ring. In the process of thermal decomposition, it can trap and burn free radicals and catalyze carbon formation [51]. MEL is a triazine compound with a nitrogen heterocyclic structure (nitrogen content up to 68%), which is a kind of typical bulk-additive nitrogen-based FR. During thermal decomposition, non-flammable gases (e.g., NH_3_ and N_2_) are released, which play the roles of dilution, heat absorption, and cooling [52,53]. MEL and PA are actively studied because of their modifiable chemical structures. The following summarizes the types of 2D SFRs designed using MEL and PA as the basic building units (Figure 5). The supramolecular self-assembly of MEL and PA occurs in the aqueous phase. There is a double synergistic effect of ion attraction (phosphate anion and -NH_3_^+^) and hydrogen bonding between PA and MEL. And the special triazine ring structure of MEL leads to π-π stacking, thus forming a 2D nanolayered structure (Figure 5a) [54,55]. The MEL-PA assembly can be obtained by simple filtration, washing and drying. Based on MEL-PA, the idea of grafting metal ions (e.g., Cu^2+^, Zn^2+^, Ni^2+^, Mg^2+^, and Mn^2+^, Figure 5b) was developed, which can improve the synergistic flame-retardant effects of MEL-PA such as cross-linking and catalytic carbonization and smoke release inhibition [56,57,58]. The metal ions mainly chelate strongly with the phosphate group structure of PA. Xiaodong Qian [57] found that Cu^2+^, Zn^2+^, and Ni^2+^ doping into MEL-PA made the 2D lamellar structure smoother. Wen Xiong Li [56] found that doping with Mn^2+^ made MEL-PA nanosheets thicker and the surface rougher. This is because during the self-assembly process, Mn^2+^ was added to the MEL-PA supramolecular structure, which affected the strength of internal interaction forces and made the assembly skeleton expand outward. In addition, based on MEL and PA raw materials, other organic or inorganic compounds were selected for multi-component self-assembly, demonstrating the flexibility of supramolecular self-assembly (Figure 5c) [58,59,60,61]. Introduced components, such as sulfanilic acid [60] and amine-functionalized AL_2_O_3_ [61], have multiple active sites and are capable of self-assembly with MEL-PA in the aqueous phase through multiple synergies such as ion attraction and hydrogen bonding. Some of the morphology and structure of the multi-component modified MEL-PA will change, and some will still show 2D sheet structure.

**Figure 6:**
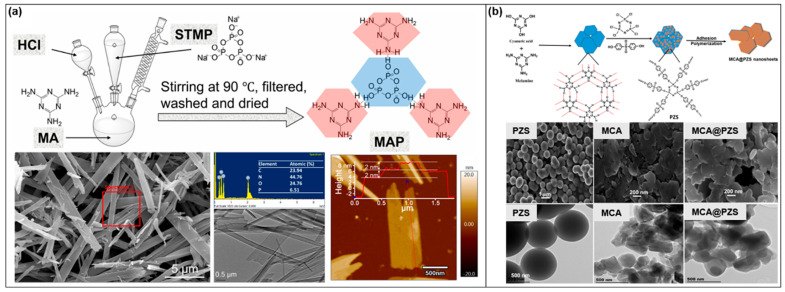
(**a**) The preparation of MAP, the corresponding micromorphology and EDS analysis [65]. (**b**) The hybridization process of MCA and PSZ, and the corresponding SEM images [64].

In addition to MEL-PA, other components containing flame-retardant elements have been selected for the self-assembly of 2D SFRs [63,64,65,66]. Most of them take advantage of multiple interactions such as electrostatic interaction, hydrogen bonding, π-π interaction, coordination between metal ions and chelating groups among different assembly units in the aqueous phase. Peifan Qin [65] selected MEL and sodium trimetaphosphate (STMP) with hydrogen bond association groups as the assembly units to construct a SFR (named MAP), based on multiple hydrogen bond interactions. MAP showed a ribbon-shaped layered structure (Figure 6a). Kuruma Malkappa [64] made full use of MEL and cyanuric acid for their phenyl-like ring structure of triazine to synthesize a 2D FR (named MCA). MCA nanosheets were obtained based on the conjugation of large π electron clouds on a benzoid ring and multiple hydrogen bonds. Dimethyl sulfone (DMSO) with high polarity was used as the solvent. Using triethylamine (TEA) as the acid-binding agent, PZS was synthesized by the substitution reaction between cyclotriphosphazene and 4,4′-sulfonyl diphenol. Then PZS first aggregated into nuclei based on hydrogen bonding and then attached to the surface of MCA. It was found that the surface of MCA nanosheets became smooth after hybridization with PZS (Figure 6b).

### 2.3. Three-Dimensional Supramolecular Flame Retardants

**Figure 7:**
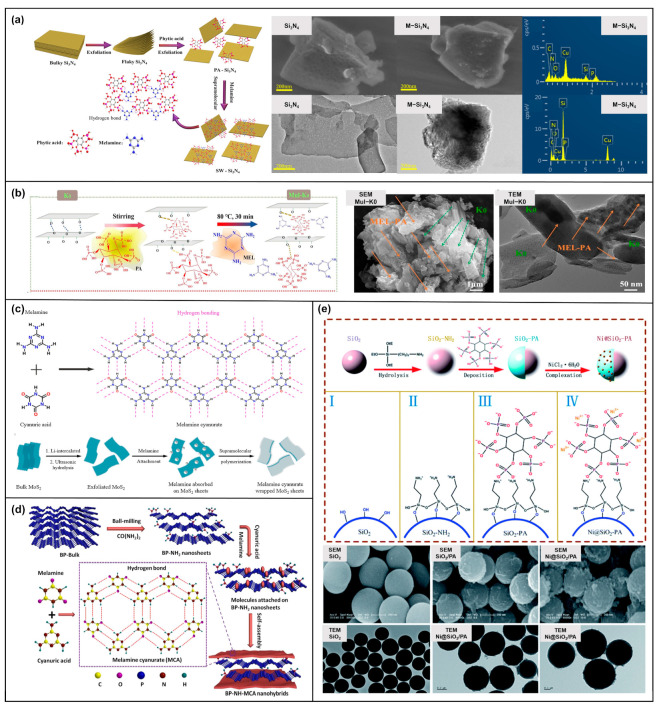
(**a**) Modification diagram of Si_3_N_4_ nanosheet; SEM, TEM and element spectra before and after modification [71]. (**b**) Multi-dimensional modification diagram of metakaolinite; SEM and TEM images after modification [72]. (**c**) The sandwich structure assembly diagram of MoS_2_ sheet and melamine cyanurate [73]. (**d**) The sandwich structure assembly diagram of aminated-BP nanosheets and melamine cyanurate [74]. (**e**) The assembling process of SiO_2_ nanosphere core-shell structure. SEM and TEM images of SiO_2_ nanospheres before and after modification [75].

The design of 3D SFRs is mainly divided into two categories. The first category is supramolecular self-assembly into sandwich structures or core-shell structures on the basis of some 2D lamellar inorganic materials. Some 2D materials, such as black phosphorus (BP) [67], layered double hydroxides (LDH) [68], graphene [69], MoS_2_ [70], etc., may face problems of poor stability, compatibility, and uneven dispersion. Therefore, 3D SFRs are assembled from these materials as templates, using some substances containing flame-retardant effects to modify them. Liang Cheng [71] used Si_3_N_4_ nanosheet as the template and introduced PA in the aqueous solution. The Si_3_N_4_ template is modified by PA based on intermolecular forces. Then, MEL was introduced, which continued to be assembled with the PA (as described earlier, the Mel-PA assembly presented a 2D lamellar structure) to build a sandwich structure. The surface of Si_3_N_4_ modified by PA-MEL changed from smooth and porous to rough, but the crystal structure remained unchanged (Figure 7a). Metakaolinite also uses this idea [72]. Due to the abundant hydroxyl groups on the surface and between layers of metakaolinite, PA was introduced based on hydrogen bonding. And then a layered stacking structure was constructed by electrostatic assembly with MEL (Figure 7b). Xiaming Feng [73] and Shuilai Qiu [74] utilized melamine cyanurate as a bridge. Using MoS_2_ sheets (Figure 7c) and aminated-BP nanosheets (Figure 7d) as the templates, respectively, the sandwich structure was self-assembled. Besides, there are core-shell structures. Yanlong Sui [75] took -NH_2_-modified SiO_2_ nanoparticles as the core, coated PA as the shell by strong electrostatic action in the mixed solution of ethanol and water, and finally introduced Ni^2+^ based on coordination. The addition of Ni^2+^ made the surface of SiO_2_@PA microspheres coarser than the smooth surface of SiO_2_ nanospheres. The microspheres were all independently dispersed. The thickness of the composite shell was about 40 nm (Figure 7e).

**Figure 8:**
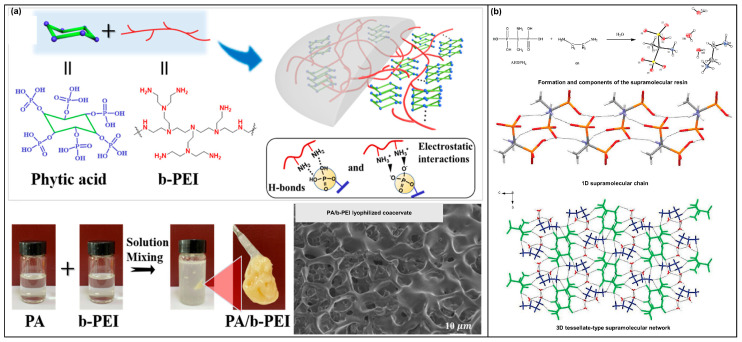
(**a**) Schematic diagram and physical diagram of PA and b-PEI assembly; SEM image of the assembled sample after lyophilization [76]. (**b**) The chemical structure of two units for assembly, and the structure diagram in the assembly process [77].

The second category is supramolecular self-assembly among small molecules, showing a 3D network structure. Most of these small molecules contain flame-retardant elements with abundant active sites. Shuitao Gao [76] used PA and branched polyethylenimide (b-PEI) as the basic units. A 3D network was assembled through hydrogen bonding and electrostatic interaction between the phosphonic acid group of PA and the amino and imino groups of b-PEI (Figure 8a). Shuo-ping Chen [77] assembled 1-aminoethyl diphosphonic acid (AEDPH4) and ethylenediamine (En) by ion attraction in aqueous solution. The specific assembly mode was: AEDPH4 transferred two hydrogen cations (H^+^) to En. At this time, there was a strong hydrogen bond between the AEDPH4 units to form a 1D zigzag chain. After receiving 2 H^+^, the En units had multiple N-H ·· O hydrogen bonds with these 1D chains. Finally, a 3D Mosaic network structure was formed (Figure 8b).

## 3. Applications of Supramolecular Flame Retardants

### 3.1. Flame Retardancy

Polymers are now used in all areas of human production and life. Polymers can be divided into plastics, fibers, and rubber, etc. [78]. Plastics are widely used due to their strong adhesion, light weight, excellent mechanical properties, and durability [79,80,81]. The typical ones are epoxy resins (EP) [74], rigid polyurethane foam (RPUF) [82], polypropylene (PP) [83], polyvinyl alcohol (PVA) [84], thermoplastic polyurethane (TPU) [85], Polyamide 6 (PA 6) [86], and poly lactic acid (PLA) [87]. In addition, textiles also penetrate all aspects of human life [88]. However, most polymers are composed of two elements, C and H. They are highly flammable. It limits the scope of their application and poses a significant fire risk. Therefore, it is very important to try various methods to make polymers stable at high temperatures and nonflammable [89,90,91]. SFRs have shown excellent effects in this regard. And they are more environmentally friendly and easier to synthesize [46,63].

#### 3.1.1. EP

**Figure 9:**
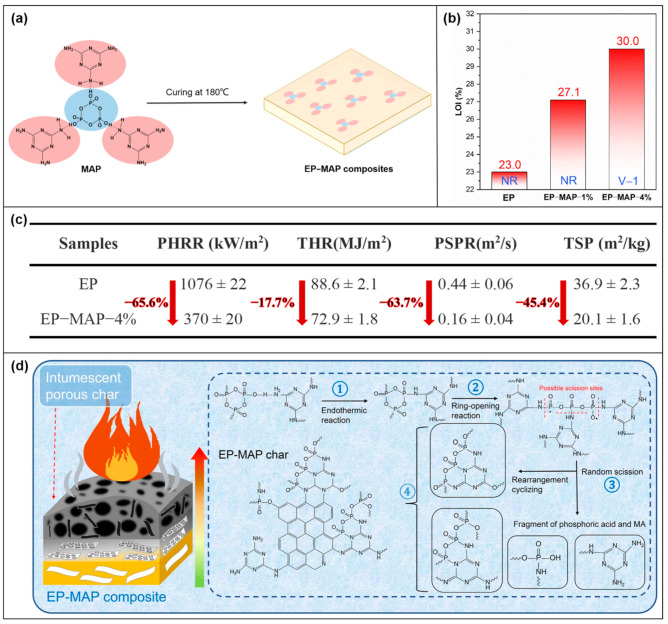
(**a**) The production route of composite materials. (**b**) Results of LOI and UL-94 tests. (**c**) CCT data: PHRR, THR, PSPR, TSP. (**d**) EP-MAP-4% carbon residue photograph. [65].

Peifan Qin et al. [65] used MAP (Figure 5a) to increase the flame retardancy of EP. MAP was added to EP by mechanical stirring at 140 °C and cured at 180 °C to produce an EP-MAP composite. The whole synthesis route and curing process are shown in Figure 9a. The flame retardancy of the EP-MAP composite was found in the vertical combustion test (UL-94) and the limiting oxygen index (LOI) test. 4 wt% MAP was added to make EP-MAP-4% composites reach a V-0 rating and the LOI value reach 30% (Figure 9b). It showed better flame retardancy than pure EP. In addition, the fire resistance of the material was further verified by the cone calorimeter test (CCT). With the addition of MAP, the peak heat release rate (PHRR) value of EP composites decreased from 1076 Kw/m^2^ to 370 Kw/m^2^, and the total heat release (THR) value decreased from 90 MJ/m^2^ to 72.9 MJ/m^2^. Figure 9c shows that the decreasing range is 65.6% and 17.7%, respectively. It proves that EP-MAP has good flame retardancy. Compared with pure EP, the peak smoke production rate (PSPR) and total smoke production (TSP) of EP-MAP composites decreased by 63.7% and 45.4%, respectively, which also proves that EP-MAP has good smoke suppression performance. The possible flame-retardant mechanism was speculated (Figure 9d), which is divided into four steps to flame retardant EP. In the first two steps, water is formed by endothermic condensation by P-O-H-N hydrogen bonds. And the vaporized MEL ring dilutes the combustible gas concentration. Inhibit the diffusion of combustible gas in the gas phase. At the same time, MAP undergoes ring-opening and cracking reactions, generating acid sources for catalytic dehydrogenation and expanding coke to promote the formation of a dense expanded carbon layer. In the last two steps, many aromatic crosslinked structures contain heterocycles of P, N, and O elements, which make the diffusion path of combustible gas tortuous, thus slowing down the heat and mass transfer rate and achieving the flame-retardant effect.

**Figure 10:**
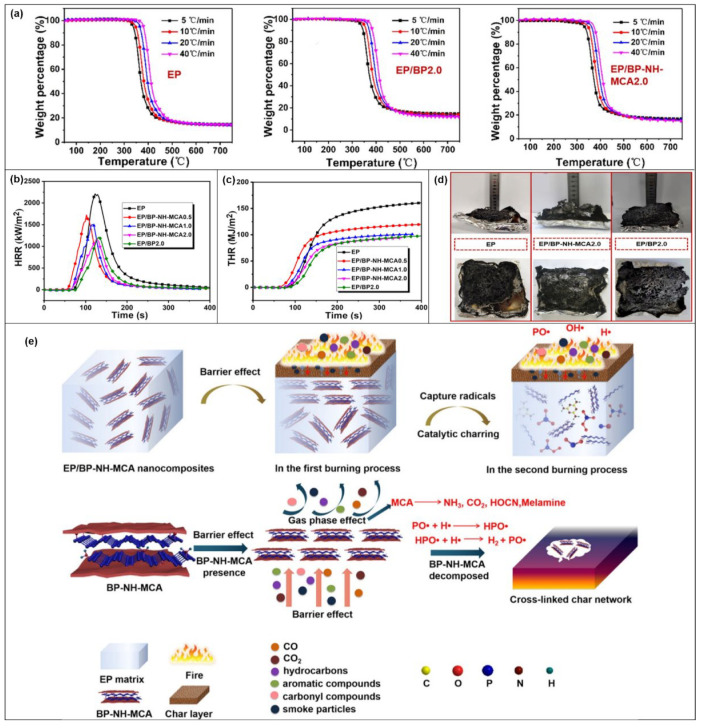
(**a**) TGA curves of EP, EPBP2.0 and EPBP-NH-MCA2.0 at different heating rates. (**b**) HRR curve of EP composites. (**c**) THR. (**d**) SPR. (**e**) TSP, Schematic diagram of flame-retardant mechanism [74].

The low loading of SFRs into the EP not only greatly improves its flame retardancy but also its thermal stability. Shuilai Qiu and co-workers [74] applied BP-NH-MCA (Figure 7d) to EP. The authors used thermogravimetry (TGA) to analyze the activation energy of thermal decomposition of pure EP and EP/BP-NH-MCA composites at different heating rates. From the data (Figure 10a), it is clear that the addition of 2 wt% BP nanosheets and 2 wt% BP-NH-MCA hybrid particles can increase the thermal degradation activation energy of EP by 11.00% and 22.85%, respectively. While the activation energy of EP/BP-NH-MCA 2.0 is 34.81% higher than that of pure EP at the maximum mass loss rate of the composites, indicating a good synergistic flame-retardant effect between BP and MCA. There is also a great improvement in the thermal stability of the composites. Compared to pure EP, the addition of 0.5 wt% to 2.0 wt% BP-NH-MCA reduces the PHRR of the composites by 21.9 to 47.2% and the THR values by 26.7 to 42.3% (Figure 10b,c). The catalytic carbonization is facilitated by the highly efficient phosphorus–nitrogen synergistic flame-retardant system constructed by BP-NH_2_ together with MCA supramolecules, resulting in a denser continuous carbon layer than EP and EP/BP2.0 (Figure 10d). The possible flame-retardant mechanism of EP/BP-NH-MCA composites can be divided into two stages (Figure 10e). In the first stage, the BP nanosheet acts as a physical barrier, effectively preventing the escape of combustible volatiles from the EP matrix. Besides, MCA is broken down and volatilizes non-flammable gases (HOCN, CO_2_, and NH_3_) to dilute the fuel and interrupt the combustion process. In the second stage, most BP is oxidized into a series of PO_x_ and phosphoric acid derivatives in the air and combined with the nitrogen structure to form a heat-stable carbon layer. At the same time, the coke network and the cross-linked phosphorus oxynitride form a physical barrier, which can delay the escape of flammable volatiles from the EP matrix.

#### 3.1.2. Rigid Polyurethane Foam

**Figure 11:**
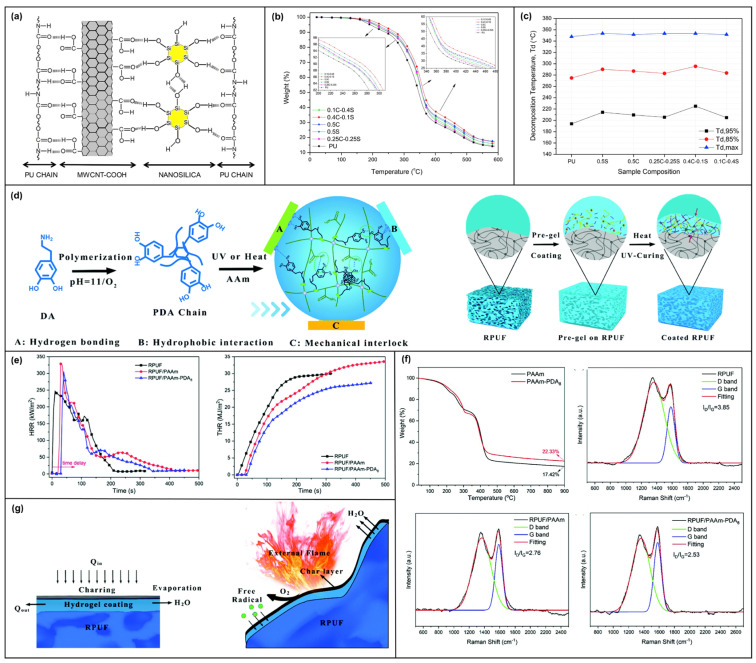
(**a**) Schematic representation of the forces between modified MWCNTs and nanosilica assemblies and RPUFs. (**b**) TGA of RPUF under different assembly conditions. (**c**) Comparison of decomposition temperature under different weightlessness. (**d**) Diagram of dual network hydrogel applied to RPUF [92]. (**e**) The HRR and THR curves of the RPUF before and after the flame-retardant coating treatment. (**f**) Raman spectra of RPUF carbon residues before and after the flame-retardant coating treatment. (**g**) Schematic diagram of the flame-retardant mechanism of RPUF treated with the coating [82].

Engin Burgaz [92] found that compared with the effects of -COOH modified MWCNTs or nanosilica applied to RPUF alone, their effects were not as good as those of binary complexes assembled based on multiple hydrogen bonding (Figure 11a). The effects of modified MWCNTs and nanosilica assemblies of different proportions on the properties of RPUF were also investigated. The results show that modified MWCNTs (0.4 wt%) and nanosilica (0.1 wt%) assemblies can improve the thermal decomposition of RPUF (Figure 11b). The thermal decomposition temperature reached its highest at 5%, 15% weightlessness and maximum mass loss (Figure 11c).

Yubin Huang [82] proposed a dual network hydrogel based on the combination of covalent and non-covalent interactions. Use the hydrogel as a flame-retardant coating for RPUF. Supramolecular-assembly was mainly manifested by multiple hydrogen bonds and π-π stacking between polyacrylate and polydopamine chains (Figure 11d). In CCT, the ignition time of pure RPUF is only 6 s. The ignition time of RPUF after the double network hydrogel coating is increased to 36 s, indicating that the fire resistance is improved. The mean HRR of RPUF with a dual network hydrogel coating is reduced by 39.7% (Figure 11e). According to Raman spectrum analysis, the residual carbon quality of RPUF coated with dual network hydrogel is the densest (Figure 11f), which can effectively play a flame-retardant role in the condensed phase. In addition, the aerogel coating evaporates water when it burns, creating a concentration that dilutes combustible gases and heat in the gas phase. It also has the function of quenching free radicals (Figure 11g).

#### 3.1.3. PP

**Figure 12:**
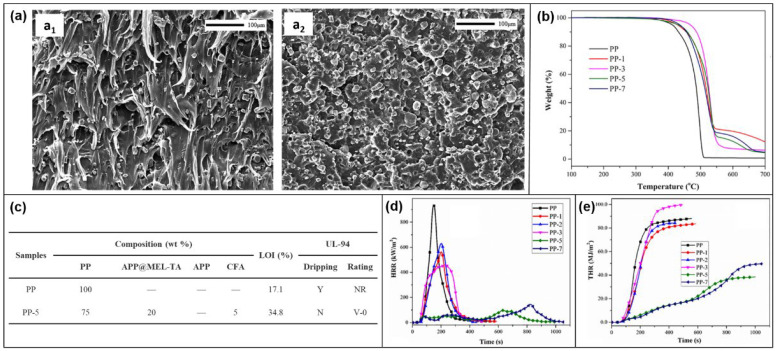
(**a**) SEM image of fracture surface: (**a_1_**) PP-7 and (**a_2_**) PP-5. (**b**) TGA curves of PP composites. (**c**) The ratio and flame-retardant test results of PP composites (**d**) HRR. (**e**) THR [93].

Congrui Qi and colleagues [93] used melamine-trimesic acid (MEL-TA) supramolecular aggregates to modify the surface of ammonium polyphosphate (APP). The modified APP and carbonated foaming agent (CFA) were then blended into the PP matrix. The combustion properties of PP composites and the corresponding flame-retardant mechanism were investigated. The cross-sectional SEM images of the PP composites are shown in Figure 12a. The dispersion effect of modified APP in PP is obviously better than that of unmodified APP, which improves the compatibility in the matrix. The TGA image (Figure 12b) also shows that the maximum thermal decomposition rate of the PP composite is significantly lower than that of pure PP, indicating that the addition of APP@MEL-TA has improved the thermal stability of PP. The ratio of PP composites and the corresponding flame-retardant performance data are shown in Figure 12c. When the mixing ratio of APP@MEL-TA and CFA is 4:1, the LOI value of PP-5 reaches its maximum value of 34.8%. In addition, PP-5 can reach the V-0 rating in the UL-94 test without dripping, while pure PP can not reach the rating with dripping. Pure PP releases a lot of heat after ignition, with a PHRR of 937 kW/m^2^ and a THR of 87.8 MJ/m^2^. The PHRR and THR values of PP-5 decreased to 97 kW/m^2^ and 38.5 MJ/m^2^, respectively (Figure 12 d,e), which further proves that the modified FR has an excellent flame-retardant effect.

#### 3.1.4. PVA

**Figure 13:**
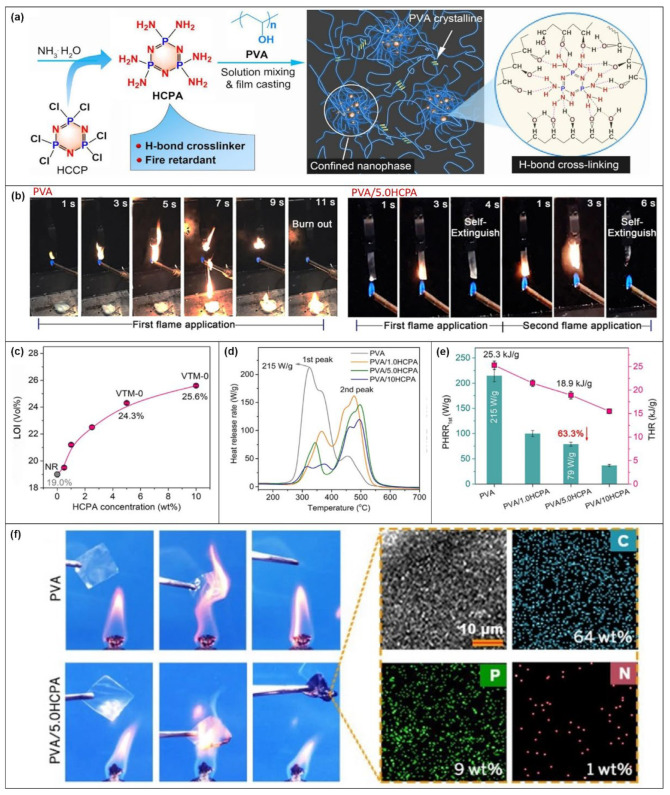
(**a**) Preparation route of PVA/HCPA composite membrane. (**b**) Digital images of PVA and PVA/5.0HCPA film vertical combustion test. (**c**) LOI test results. (**d**) HRR of MCC. (**e**) PHRR and THR values. (**f**) Composite film combustion image and SEM-EDS image of residual carbon after PVA/5.0HCPA film combustion. [94].

Lei Liu et al. [94] designed and synthesized a polyamine small molecule (named HCPA) that can be used as a FR for PVA. The flame-retardant effects of different HCPA concentrations on PVA were investigated (Figure 13a). UL-94 and LOI measurements were used to evaluate the flame retardancy of the film. Pure PVA film burns rapidly within 12 s of ignition, accompanied by heavy dripping, resulting in failure to reach the rating (Figure 13b). However, the PVA/5.0HCPA film rapidly self-extinguishes after the first ignition and continues to burn for only 3 s after the second ignition, during which no molten drops are observed, thus achieving the required UL-94 VTM V-0 rating (Figure 13c). The addition of 5.0 wt% HCPA increases the LOI of the original PVA from 19.0% to 24.3%. Similarly, with the increase in HCPA content to 10 wt%, the LOI value will increase to 25.6% (Figure 13c).

Figure 13d,e shows the test data from the Micro Cone Calorimetry (MCC) method. The first PHRR value of PVA/5.0HCPA film decreases by 63% compared with the original PVA. The THR value decreases from 25.3 kJ/g to 18.9 kJ/g. Furthermore, adding PVA/10HCPA can reduce the THR value by 38.5%. The decrease in these flame-retardant parameters confirms that HCPA improves the flame retardancy of PVA. After the PVA/5.0HCPA composite film is ignited by an alcohol lamp, it leaves a complete and dense carbon residue, which is in sharp contrast with the original PVA with no residue (Figure 13f), reflecting the good carbonization ability of HCPA. Therefore, PVA/HCPA can achieve a synergistic flame-retardant effect in the gas phase and condensed phase.

#### 3.1.5. TPU

**Figure 14:**
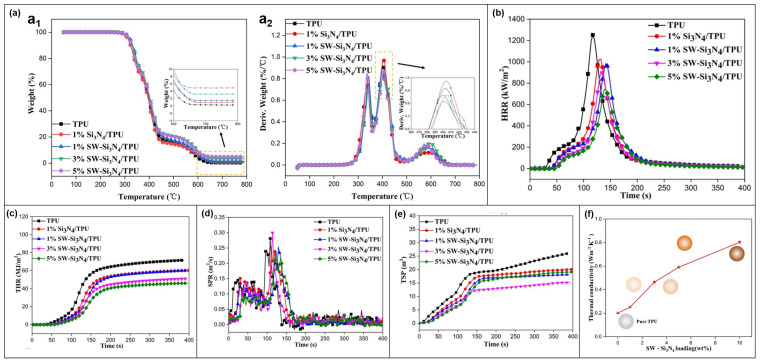
(**a**) Data curves: (**a_1_**) TGA and (**a_2_**) DTG. (**b**) HRR. (**c**) THR. (**d**) SPR. (**e**) TSP. (**f**) Thermal conductivity of TPU composites [71].

Liang Cheng et al. [71] proposed SW-Si_3_N_4_ (Figure 7a), which can effectively reduce the fire risk of TPU materials. The TGA analysis curves are shown in Figure 14a. The addition of 5 wt% SW-Si_3_N_4_ generates 4.97 wt% of residual carbon at 800 °C in SW-Si_3_N_4_/TPU, which is higher than the 0.30 wt% of residual carbon in pure TPU. In addition, the SW-Si_3_N_4_ hybrid nanosheets in the TPU matrix are able to suppress heat transfer more effectively compared to the Si_3_N_4_ nanomaterials. The combustion behavior of the TPU composites was investigated by CCT. Figure 14b,c shows the HRR and THR curves. Figure 14d,e shows the data curves of SPR and TSP. It is obvious that the smoke release is inhibited by each doping amount of TPU composites, which also confirms the good smoke suppression property of SW-Si_3_N_4_.

The thermal conductivity of SW-Si_3_N_4_/TPU (Figure 14f) shows that the thermal conductivity increases with increasing filler content and reaches approximately a fourfold increase in 10% SW-Si_3_N_4_/TPU. The TPU composite has an internal heat transfer path. It is conducive to heat diffusion and can reduce the risk of fire due to heat buildup locally in the matrix.

#### 3.1.6. PA6

**Figure 15:**
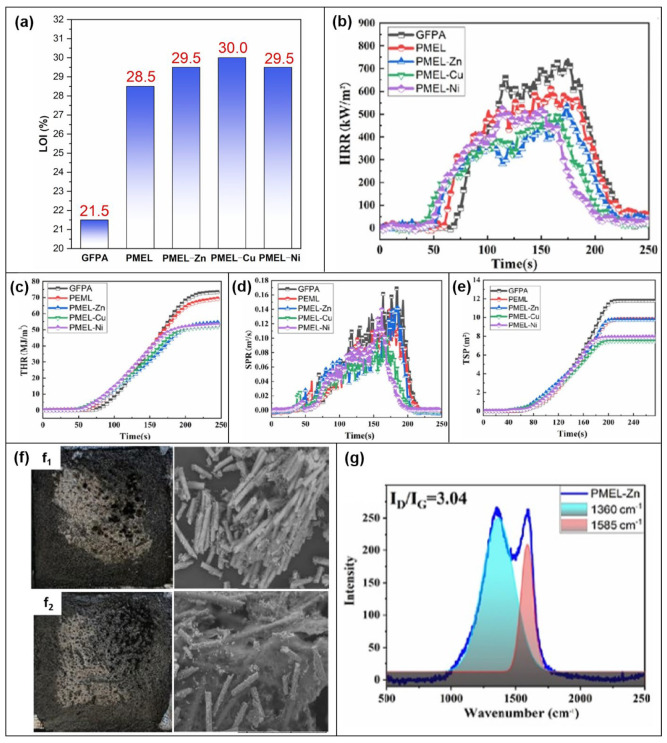
(**a**) Ratio of PA composite and LOI values. (**b**) HRR. (**c**) THR. (**d**) SPR. (**e**) TSP. (**f**) SEM image of carbon residue: (**f_1_**) PA-MEL and (**f_2_**) PA-MEL-Cu. (**g**) Raman image of PA-MEL-Zn. [57].

Xiaodong Qian et al. [57] studied the effect of PA-MEL with metal ions grafted (Figure 5b) on the properties of PA6. The LOI value of the composite increases from 21.5% to 30.0% after the addition of PA-MEL-phosphate/transition metal (Figure 15a), demonstrating the good flame-retardant effect. The flame-retardant properties of the composites were further evaluated by CCT. Pure glass fiber reinforced PA6 (GFPA) burns rapidly after ignition and has a PHRR value of up to 739.97 kW/m^2^. The addition of PA-MEL reduces the PHRR of the composite to 612.38 kW/m^2^. In particular, the addition of PA-MEL-Cu leads to a reduction of up to 32.37% in the PHRR value (Figure 15b). Moreover, the THR value of the PA-MEL-Cu composite decreases by 28.04% compared with GFPA (Figure 15c). All SPR peaks for the composites are lower than GFPA after the addition of the PA-MEL-phosphate or transition metal (Figure 15d). The TSP of the GFPA/PA-MEL-Cu composites is reduced by up to 36.7% (Figure 15e). Compared with unmodified PA-MEL, the addition of PA-MEL-Zn will increase the number of carbon layers (Figure 15f) and the degree of graphitization of the residual char (Figure 15g). A possible flame-retardant mechanism was proposed. PA promotes the formation of pyrophosphate or polyphosphate and catalyzes the formation of a stable carbon layer. MEL releases non-combustible gases such as NH_3_ and H_2_O to achieve dilution. In addition, the incorporation of transition metals has a synergistic flame-retardant effect, which can promote the formation of dense carbon layers.

#### 3.1.7. PLA

**Figure 16:**
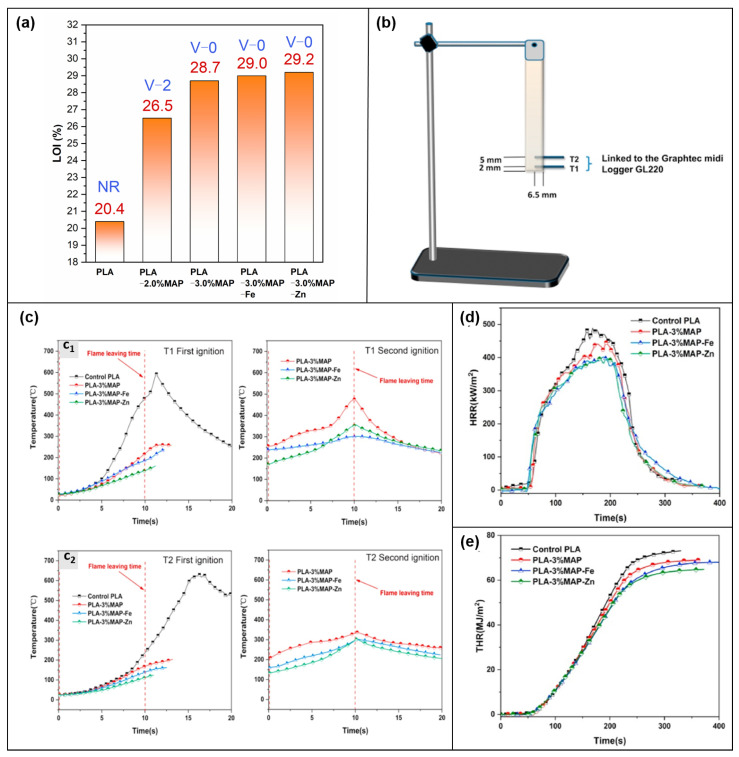
(**a**) Test results of LOI and UL-94. (**b**) Diagram of internal temperature test. (**c**) Temperature data recorded by thermocouple: (**c_1_**) T1 and (**c_2_**) T2, (**d**) HRR. (**e**) THR. [58].

Qinyong Liu et al. [58] self-assembled MEL, paminobenzene sulfonic acid (ASA), and PA into a new SFR (named MAP) by a simple green method. The product after chelation with Fe^3+^ or Zn^2+^ was named MAP-Fe and MAP-Zn (Figure 5c) and was applied to PLA. The LOI value of pure PLA is only 20.4%, which not only fails to achieve a rating in the UL-94 test but is accompanied by severe dripping. In contrast, the PLA composite achieves a V-2 rating for PLA-2% MAP while also shortening the burning time after two ignitions. PLA-3% MAP-Zn even achieves a V-0 rating, minimizing the burning time and increasing the LOI value to 29.2% (Figure 16a). Interestingly, the authors used two thermocouples (T1 and T2) to record the temperature changes at two locations during the UL-94 test (Figure 16b). The internal temperature of the PLA composite significantly reduces. Figure 16c shows the temperature profiles for each of the two ignitions. At the end of the first ignition for 10 s, T1 and T2 are reduced to 139 °C and 113 °C for 3.0% MAP-Zn, respectively (483 °C for pure PLA). The values for pure PLA are 490.1 kW/m^2^ and 73.3 MJ/m^2^, respectively, while PLA-3.0% MAP-Zn shows the lowest PHRR and THR values of 398.5 kW/m^2^ and 65.6 MJ/m^2^ (Figure 16d,e). The possible flame-retardant mechanism of PLA composites was summarized. Firstly, MAP releases NH_3_ and phosphorus-containing radicals in the gas phase to dilute oxygen with combustible volatiles while trapping H· and OH· during combustion, interrupting the chain reaction of combustion. In the condensed phase, further cross-linking of MEL and ASA will form a stable carbon layer, while Fe^3+^ and Zn^2+^ will optimize the existence of the carbon layer.

#### 3.1.8. Cotton Fabrics

Cotton fabrics are considered to be one of the most popular natural fibers due to their perspiration absorption, renewable nature, and comfortable feel [95,96]. However, cotton fabric is flammable. And the flame will spread rapidly after being ignited, thus causing a fire [97,98]. In the method of flame-retardant treatment of textiles, the most typical method is to assemble the coating layer by layer (LBL). The self-assembly of LBL makes the layers have attractive forces such as electrostatic attraction, hydrogen bonding and coordination bonds, forming supramolecular coatings, thus giving textiles flame retardancy [99,100].

**Figure 17:**
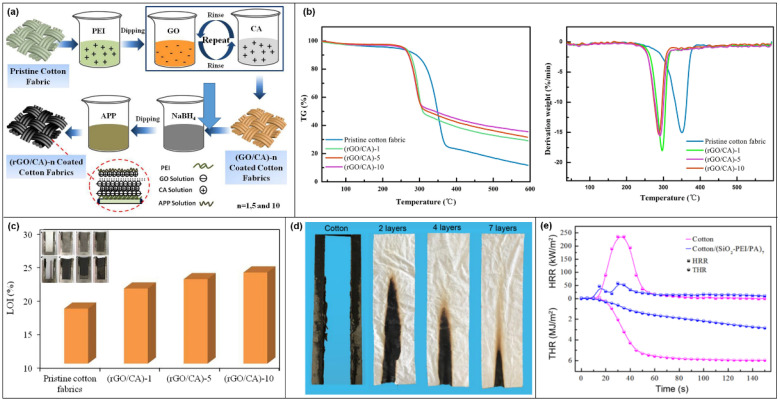
(**a**) Diagram of LBL assembly coating cotton fabric. (**b**) TGA and DTG curves of pure cotton fabrics and coated cotton fabrics with different cycles. (**c**) LOI test values [101]. (**d**) Digital images of vertical flammability test. (**e**) HRR and THR curves of cotton and cotton/(SiO_2_-PEI/PA)_7_ [102].

Wen An et al. [101] constructed flame-retardant and antistatic fabrics by LBL assembly, using pure cotton fabric as the backbone and dipping the cotton fabric alternately into cationic casein (CA) and anionic graphene oxide (GO) solutions (Figure 17a). The LBL-assembled coated cotton fabrics form the carbon layer earlier in the process of combustion. A comparison of the carbon residues at 600°C shows that the thermal stability of the cotton fabric increases with the number of assembled layers on the surface of the fabric (Figure 17b). The progressively higher LOI values (Figure 17c) also indicate that the flame resistance of the coated cotton fabric increases with the assembled layer number of rGO/CA.

Shanshan Li et al. [102] designed a novel organic-inorganic hybrid intumescent flame-retardant coating by using nanosilica (SiO_2_) covered with polyethyleneimine (PEI) and PA. It was applied to the surface of cotton fabric by LBL assembly. It achieved a good flame-retardant effect with only 7 bilayers. The UL-94 test shows that compared with pure cotton fabric, the combustion of coated cotton fabric is relatively slow. The spread of flame from the bottom of the coated cotton fabric is gradually weakened (Figure 17d). The PHRR of Cotton/(SiO_2_-PEI/PA)_7_ is 58 kW/m^2^. It is 75% lower than that of untreated cotton fabric. The coating assembled by LBL within 150 s reduced the THR value of cotton fabric from 5.94 MJ/m^2^ to 2.83 MJ/m^2^ (Figure 17e).

### 3.2. Mechanical Properties

As we all know, in order to achieve efficient flame retardancy, the loss of mechanical properties is inevitable [103,104]. The successful preparation of SFRs has made great contributions to achieving flame retardancy and minimizing the loss of mechanical properties [46,105].

**Figure 18:**
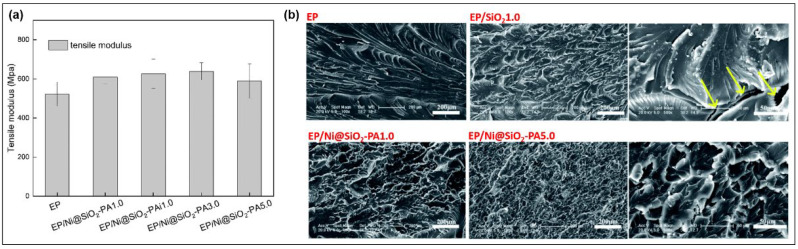
(**a**) Tensile modulus diagram. (**b**) SEM images of EP and EP composites [75].

A new green FR, Ni@SiO_2_-PA (Figure 7e), has been applied to flame retardant EP. The strength of EP nanocomposites has been significantly improved. This core-shell FR was made by Yanlong Sui et al. [75] The tensile modulus of EP and its composites is shown in Figure 18a. Due to the strong hydrogen bonding, there is a strong interface interaction between Ni@SiO_2_PA and the EP matrix, which makes the tensile modulus of EP/Ni@SiO_2_-PA3.0 higher than that of pure EP by 22.2%. Compared with the smooth surface of pure EP, large pores form in the EP/SiO_2_1.0 composite with the obvious agglomeration of SiO_2_ particles. EP/Ni@SiO_2_-PA1.0 and EP/Ni@SiO_2_-PA5.0 composites show a rough and inhomogeneous fracture surface (Figure 18b), demonstrating the extremely high supramolecular shell-matrix compatibility.

**Figure 19:**
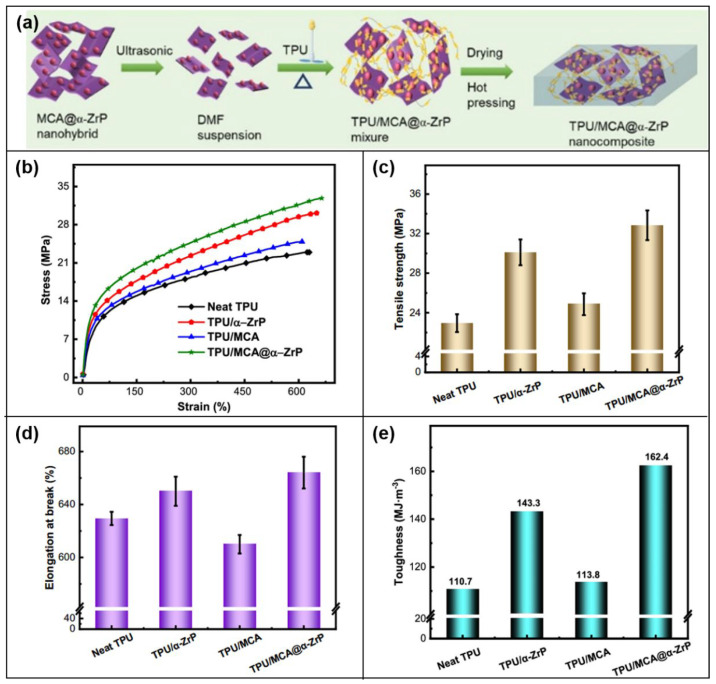
(**a**) Preparation of TPU/MCA@α-ZrP nanocomposites. (**b**) Stress-strain curves of TPU and its composites. (**c**) Tensile strength histogram. (**d**) Elongation at break histogram. (**e**) Fracture energy data image [106].

Melamine cyanorate/α-ZRP nanosheets (MCA @ α-ZRP) [106], also made by supramolecular assembly technology, were applied to TPU (Figure 19a). And H· was used to strengthen the interface with the TPU matrix and limit the fluidity of the polymer chain, thus successfully avoiding the decrease of mechanical properties with the addition of FRs. As shown in Figure 19b, TPU composites all show better mechanical properties than pure TPU. TPU/MCA@α-ZrP increases the tensile strength by 43.1% at the maximum (Figure 19c). There is a synergistic effect in the MCA@α-ZrP blend, which reduces the influence of MCA alone on the flexibility of TPU (Figure 19d) and increases the fracture strain of TPU composites from 629% to 664%. Besides, this study calculates the fracture energy of the TPU composites in order to indirectly estimate their toughness (Figure 19e). The hydrogen bond breakage between the MCA@α-ZrP blend and TPU, leads to the reconstruction of the H-bond, inducing stress transfer and ductility, which consume more fracture energy. Therefore, TPU/MCA@α-ZrP shows the highest fracture energy of 162 MJ/m^3^.

**Figure 20:**
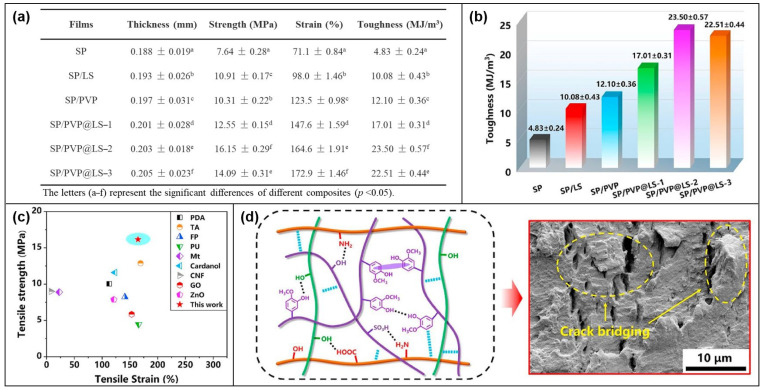
(**a**) Data on the mechanical properties of SP and its composites. (**b**) Toughness of the materials. (**c**) Comparison of the strength and flexibility of SP/PVP@LS films with other films. (**d**) Illustration of possible toughening mechanism analysis [107].

Kuang Li et al. [107] prepared soy protein (SP)-based films (PVP@LS) with supramolecular network structures including dynamic H, π-π interactions and interconnected water transport interactions, which can effectively dissipate energy when the films are stretched. These non-covalent interactions are beneficial to improve the adhesion and cohesion of the films so that they can have high tenacity and tensile strength along with high flame retardancy. As shown in Figure 20a,b, the tensile strength and toughness of SP/PVP@LS-2 film are 111.39% and 386.54% higher than those of pure SP film, respectively. The peak values are as high as 16.15 MPa and 23.50 MJ/m^3^. In order to further prove the enhancement of the mechanical properties of the SP composite film, the author compared the strength and elongation at break of the SP composite film with other reports. SP/PVP@LS shows better tensile strength (Figure 20c). Figure 20d shows a possible toughening mechanism for SP/PVP@LS films, showing the microscopic and macroscopic mechanisms of the SP composite film under external loading stress, respectively. This in turn demonstrates the toughening effect of a strong and stable supramolecular crosslinking network on the SP composite film.

### 3.3. Other Properties

In fact, all kinds of electronic equipment, furniture, clothing, etc. used in our lives are easy to burn after encountering high temperatures or an open flame [108]. As a guarantee of fire safety, FRs can solve the burning problem. But it will greatly reduce the excellent performance of the original material [109]. The advantage of supramolecular structures formed by various non-covalent interactions is their high compatibility with the material. SFRs can play a flame-retardant role without destroying other special properties of the material. And they can even play a role in gaining. Therefore, researchers focus on the effects of supramolecular structure on the thermal insulation, self-healing, and UV-blocking of flame-retardant materials. In addition, there are also studies on improving the electrochemical properties of batteries [110,111,112], the viscoelasticity of thermoplastic materials [113] and the flame-retardant durability of fibrous materials [114].

#### 3.3.1. Thermal Insulation Performance

Comfortable indoor temperatures have always been a concern for people. A lot of energy is needed to achieve this goal. So heat insulation materials with high heat insulation performance are very important [115]. However, most insulation materials are flammable, and the addition of FRs will destroy the insulation performance. But the addition of SFRs can reduce the reduction in insulation performance of many combustible insulation materials while achieving a high level of flame retardancy [60,105].

**Figure 21:**
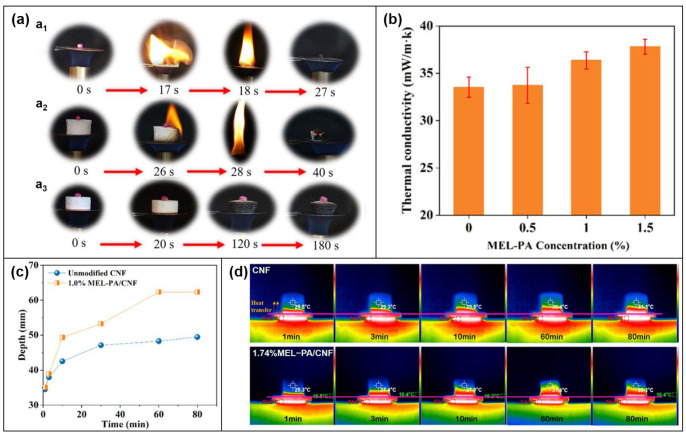
(**a**) Screenshot of insulation inspection test: (**a_1_**) match end, (**a_2_**) Place the match head in the unmodified CNF aerogel and (**a_3_**) Match heads placed in 1.0%MEL-PA/CNF composite aerogel. (**b**) Thermal conductivity of MEL-PA/CNF composite aerogels. (**c**) Thermal penetration depth of aerogel before and after modification. (**d**) Thermal infrared image of CNF and 1.74%MEL-PA/CNF [105].

Xueyong Ren et al. [105] modified the gas coagulation of cellulose nanofibril (CNF) by in-situ supramolecular assembly of MEL-PA. A simple simulation experiment was designed to test the thermal insulation ability of the MEL-PA/CNF composite aerogel. As shown in Figure 21a, when the match head was placed on the top of the modified MEL-PA/CNF composite aerogel, neither the composite aerogel nor the match head ignited, but the match head on the top of the unmodified CNF aerogel ignited. When the aerogel burns for 28 s, which not only shows the flame-retardant effect of the MEL-PA/CNF composite aerogel but also shows the thermal insulation effect. To further demonstrate the thermal insulation properties of the composite aerogels, the authors tested their thermal conductivity and observed their heat transfer using an infrared camera. The thermal conductivity of the MEL-PA CNF aerogel is relatively higher due to the enhanced interfacial interaction between the MEL-PA and CNF aerogels (Figure 21b). But the thermal propagation curve (Figure 21c) shows that the heat transfer could reach a relatively stable state after 60 min. And the infrared thermography (Figure 21d) similarly shows that the heat transfer can be effectively blocked before and after the modification. From the above analysis, this work concluded that the supramolecularly-assembled MEL-PA does not significantly deteriorate the thermal insulation of CNF aerogels.

#### 3.3.2. Self-Healing Property

Self-healing behavior contributes to the recycling of materials, helping to achieve sustainability and the rapid development of recycled polymers [116,117]. The presence of reversible non-covalent bonds imparts dynamic structural properties to the material, hence the self-healing properties of supramolecular materials [63,118].

**Figure 22:**
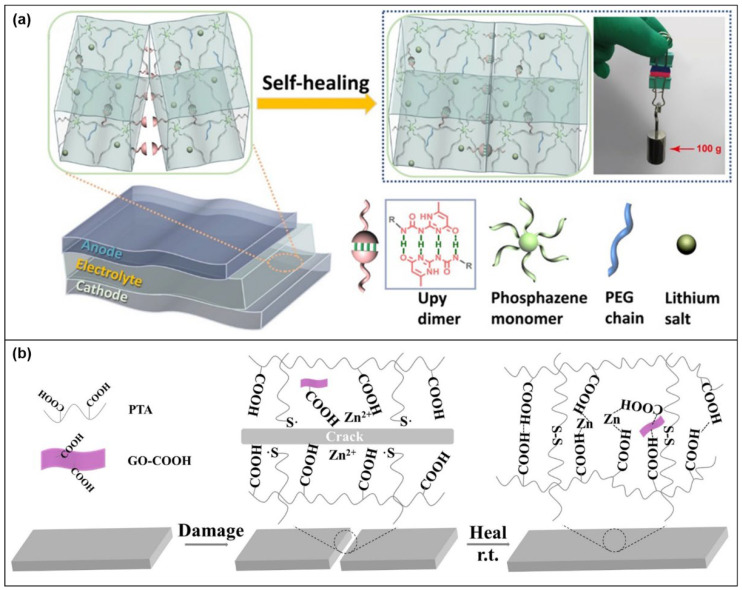
(**a**) Schematic diagram of CPSHPEs self-healing mechanism [118]. (**b**) Diagram of self-healing mechanism of other dynamic keys [63].

The cyclic phosphonitrile-based polymer electrolyte (CPSHPE) prepared by Binghua Zhou et al. [118] has self-healing properties. Figure 22a shows the self-healing mechanism of CPSHPEs. The authors cut the CPSHPE into two pieces and found the presence of dynamic hydrogen bonds at the cuts, which gave the material self-healing properties due to the tendency of such non-conjugated hydrogen bonds to combine to form supramolecular networks. In addition to hydrogen bonding, there is an abundance of dynamic bonds such as dynamic disulfide bonds and ionic coordination bonds (Figure 22b) [63]. It can cause damage to generate free dynamic groups that can reassemble into a stable cross-linked network when the damaged area comes into contact again, thus restoring the material to its original properties.

#### 3.3.3. UV-Blocking Performance

Ultraviolet (UV) radiation has attracted extensive attention because of its negative effects on mechanical properties, discoloration, and decomposition of materials [119,120]. Compared with the flame-retardant polymer with intumescent flame retardant (IFR), SFRs can avoid accelerating the aging of the substrate under ultraviolet radiation or heating, thus reducing the flame-retardant performance of the polymer. At the same time, the degradation of mechanical properties is reduced, with excellent ultraviolet resistancy [107].

**Figure 23:**
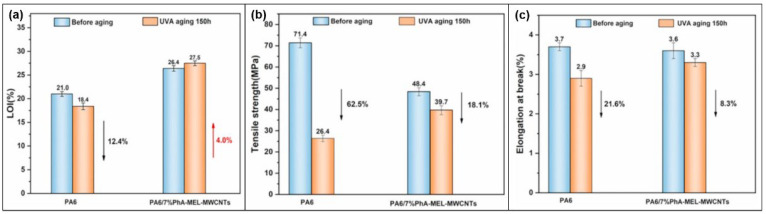
(**a**) Comparison of LOI values of PA6 and PA6/7% MEL-PA-MWCNTs after UV aging. (**b**) Comparison of tensile strength and (**c**) elongation at break after UV aging 150h [46].

MEL has a special triazine ring structure, so it can absorb ultraviolet rays and show anti-aging performance. Yuchun Li et al. [46] developed MEL-PA-MWCNTs (Figure 3c), which were applied to flame retardant PA6 and PA6 composites, showing good ultraviolet resistance. The LOI value of PA6/7% MEL-PA-MWCNTs does not decrease but increased after photoaging (Figure 23a). Mainly because MEL-PA-MWCNTs protect the substrate and migrated to the surface of the substrate under the irradiation of ultraviolet light, thus avoiding the aging and burning of PA6. The tensile strength and elongation at break of PA6 composites are much lower than those of pure PA6 (Figure 23b,c), due to the absorption of ultraviolet radiation by triazine ring groups in MEL and the capture of free radicals produced by PA in the aging process of the matrix.

## 4. Conclusions and Prospect

SFRs of different dimensions (including 1D, 2D, and 3D SFRs) show excellent flame-retardant efficiency in improving the fire safety of typical polymers. Similar to the flame-retardant mechanism of traditional FRs, SFRs also mainly plays a role in the gas phase and condensed phase. On the one hand, SFRs containing typical flame-retardant elements such as phosphorus and nitrogen can interfere with the free radical reaction during combustion and slow down the combustion rate. On the other hand, they can help to char yield, which acts as a heat transfer barrier. Meanwhile, the 2D and 3D structural characteristics of SFRs are conducive to blocking the transfer of O_2_, flammable volatile substances, and heat.

SFRs have a good effect on improving the properties of polymers. In contrast to traditional FRs synthesis methods, SFRs are mostly self-assembled by using different primitives in non-toxic and pollution-free solvents (water or ethanol). It mainly utilizes the multiple synergies of hydrogen bonding, ion attraction, π-π stacking, and other non-covalent bonds among the units. These units usually contain groups rich in active sites, such as amino, phosphate, and hydroxyl groups. However, the selection of units is relatively limited, such as PA and MEL, which have been widely studied. More units containing flame-retardant elements with multiple active sites should be dug out for design and assembly. The application prospects of novel SFRs should be explored. At the same time, compared with some other FRs applied to the matrix in the form of simple physical dispersion, SFRs have rich active groups, can produce some crosslinking (such as multiple hydrogen bonds). They have interface interactions with the matrix and have good compatibility. Therefore, the mechanical properties of the polymer are improved.

In addition, on the basis of the improvement in fire safety performance, the performance of SFRs on other aspects of the matrix has been improved synchronously, such as ultraviolet protection, self-healing, electrochemical performance, and other properties. This is a good research idea for the high-performance requirements of materials now. At present, there are few such studies, and continuous research work is needed. In particular, the self-healing performance of SFRs is worth studying. SFRs, based on the dynamic reversible non-covalent bond synthesis of structural characteristics, should be made full use of. Under the current ecological background, the recyclability, repairability, degradability and recycling of polymers will be vital topics.

## Data Availability

Not applicable.
